# Different Aspects of Aging in Migraine

**DOI:** 10.14336/AD.2023.0313

**Published:** 2023-12-01

**Authors:** Michal Fila, Elzbieta Pawlowska, Joanna Szczepanska, Janusz Blasiak

**Affiliations:** ^1^Department of Developmental Neurology and Epileptology, Polish Mother’s Memorial Hospital Research Institute, 93-338 Lodz, Poland.; ^2^Department of Pediatric Dentistry, Medical University of Lodz, 92-216 Lodz, Poland.; ^3^Department of Molecular Genetics, University of Lodz, Pomorska 141/143, 90-236, Lodz, Poland.

**Keywords:** migraine, chronological aging, biological aging, brain aging, social aging, cognitive aging, metabolic aging, molecular markers of aging, cellular senescence, stem cell exhaustion

## Abstract

Migraine is a common neurological disease displaying an unusual dependence on age. For most patients, the peak intensity of migraine headaches occurs in 20s and lasts until 40s, but then headache attacks become less intense, occur less frequently and the disease is more responsive to therapy. This relationship is valid in both females and males, although the prevalence of migraine in the former is 2-4 times greater than the latter. Recent concepts present migraine not only as a pathological event, but rather as a part of evolutionary adaptive response to protect organism against consequences of stress-induced brain energy deficit. However, these concepts do not fully explain that unusual dependence of migraine prevalence on age. Many aspects of aging, both molecular/cellular and social/cognitive, are interwound in migraine pathogenesis, but they neither explain why only some persons are affected by migraine, nor suggest any causal relationship. In this narrative/hypothesis review we present information on associations of migraine with chronological aging, brain aging, cellular senescence, stem cell exhaustion as well as social, cognitive, epigenetic, and metabolic aging. We also underline the role of oxidative stress in these associations. We hypothesize that migraine affects only individuals who have inborn, genetic/epigenetic, or acquired (traumas, shocks or complexes) migraine predispositions. These predispositions weakly depend on age and affected individuals are more prone to migraine triggers than others. Although the triggers can be related to many aspects of aging, social aging may play a particularly important role as the prevalence of its associated stress has a similar age-dependence as the prevalence of migraine. Moreover, social aging was shown to be associated with oxidative stress, important in many aspects of aging. In perspective, molecular mechanisms underlying social aging should be further explored and related to migraine with a closer association with migraine predisposition and difference in prevalence by sex.

## 1. Introduction

The prevalence of most human disorders increases with chronological age, but several diseases, including some kinds of cancer, peak in juvenile and adolescence [1, 2]. This supports the thesis that true aging rate is associated with biological rather than chronological aging [3]. Moreover, the occurrence of certain diseases at young age suggests that biological aging may not only differ from its chronological counterpart, but also different organs and tissues may age at different rates. These organ-specific rates of aging, along with other aspects of aging, contribute to complete aging-related characteristic of an individual called “ageotype” [4].

Migraine is a complex, chronic neurological disease with largely unknown mechanisms of pathogenesis. This gap in knowledge results from a restricted accessibility to the live human brain and limited value of animal models of human migraine [5]. Although targeting calcitonin gene related peptide (CGRP) and its receptor with antibodies and antagonists revolutionized migraine therapy, some questions on the long-term application of anti-CGRP treatment, especially in children, adolescent and the elderly are still open [6]. However, the basic question is why some patients do not response to anti-CGRP therapy. Equally important is the question why some people develop migraine and others do not.

Clinical picture of migraine dependence on age is unusual as the disease prevalence peaks between the ages of 20 and 40 years and the intensity of headache attacks decreases and responsiveness to therapy increases with age [7]. Migraine is the mean reason of disability in young women [8]. Therefore, migraine has the greatest effect when people are professionally and socially most active [9]. Therefore, it is important to understand how specific aspects of aging may influence migraine pathogenesis and vice versa - how migraine may influence the process of aging.

**Figure 1.**
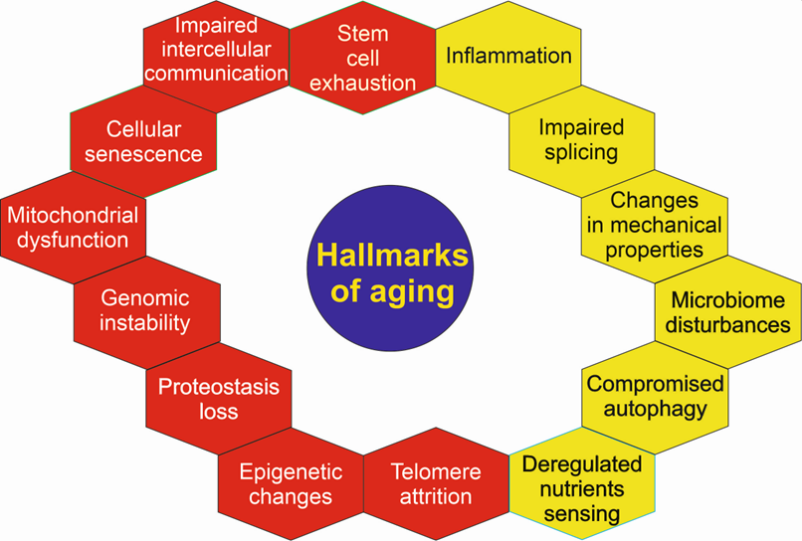
**Original (red) and new (yellow) molecular hallmarks of aging**. These hallmarks were adopted by international panels of experts and serve to identify targets in anti-aging strategies that provide precise mechanisms of aging [10]. This set of hallmarks is not complete and many of them overlap, e.g., genomic instability can be mechanistically linked to all remaining hallmarks, like epigenetic changes. Therefore, they may be considered as non-specific indicators of age as well as factors contributing to both physiological and premature aging.

Aging leads to progressive loss of homeostasis, which is at the heart of age-related health outcomes [11]. Age-related loss of homeostasis is underlined by cellular and molecular processes also known as molecular hallmarks of aging [12]. They can be divided into three categories: primary hallmarks (genomic instability, telomere erosion, changes in the epigenetic profile, and loss of proteostasis), antagonistic hallmarks (deregulated nutrient sensing, mitochondrial dysfunction, and cellular senescence), and integrative hallmarks (stem cell depletion, and altered intercellular communication). However, this set of markers is not sufficient to explain all aspects of aging and its consequences [13]. In a 2022 research symposium “New Hallmarks of Ageing” held in Copenhagen, Denmark, these hallmarks were revised and supplemented by five new ones [10] (Fig. 1). They are compromised autophagy, microbiome disturbance, altered mechanical properties, splicing dysregulation and inflammation. It is suggested that these hallmarks should be considered in an integrative form, instead of being analyzed individually to help to assess biological aging. However, we are surprised that there is not a metabolic marker among these markers. We will address that problem in subsequent paragraphs.

Many reports address aspects of aging that are not directly linked with biological markers of aging. They are usually divided into psychological and social aging. The following social determinants (hallmarks) of aging are considered: low socioeconomic status, minority status, adverse life events, adverse psychological states, and adverse behaviors [14]. Like biological hallmarks, these determinants form a network of integrated and mutually dependent features that collectively characterize the process of social aging. The concept of cognitive aging has been developed to describe a process of gradual, longitudinal changes in cognitive functions that accompany the aging process [15]. The term metabolic aging may be understood as progressive decline in metabolic rate with age and integrates some aspects of biological hallmarks of aging, first deregulation of nutrients sensing, mitochondrial impairment and declined proteostasis. All molecular hallmarks of aging and above-mentioned aspects of aging depend on genetic constitution and epigenetic profile. However, the genome sequence changes slowly, if at all, in response to environmental changes and thus epigenetic aging and epigenetic clocks are considered [16-18].

In this narrative review/perspective we present some general aspects of migraine and its connections with brain aging, hallmarks of biological aging: cellular senescence, epigenetic alterations, and inflammation as well as cognitive, social, and metabolic aging. As oxidative stress is involved in almost all, if not all, of these phenomena, its role in migraine in the context of aging is also included. Some potential implications of these connections for migraine management, especially in the elderly, are also presented.

## 2. Migraine

Migraine is a neurological disorder featured by throbbing or pulsing headache attacks and such syndromes such as vomiting, nausea, hypersensitivity to noise, light and other environmental stimuli as well as mood changes from depression to euphoria [19].

**Figure 2.**
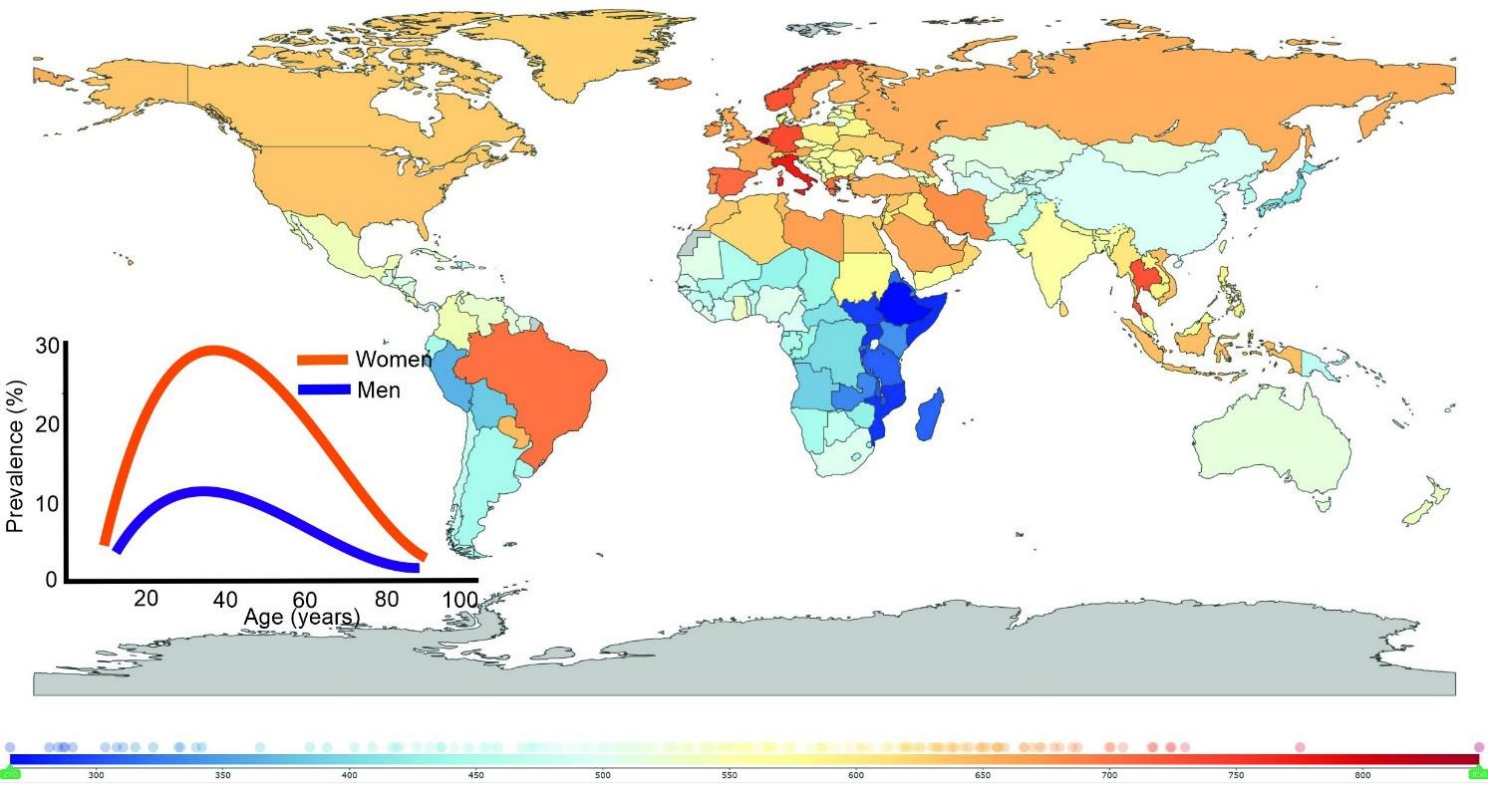
**Years lived with disability (YLD) caused by headaches in 2019 according to WHO (both sexes, all ages; https://vizhub.healthdata.org/gbd-compare/)**. The numbers below the colored scale correspond to YLDs per 100,000; inset - illustrative migraine prevalence by age and sex.

Migraine affects over one billion individuals worldwide with the highest prevalence in young adults and is a serious burden for affected individuals and societies (reviewed in [5]) (Fig. 2). Annual loss of productivity due to migraine prevalence in the United States is estimated for more than USD 20 billion [20]. It is the second cause of disability worldwide [8].

Migraine prevalence in women is 2-3 times higher than in men and this difference has not been rationally explained yet, although some studies suggest that hormonal or X-linked components as well as some mitochondrial pathways can contribute to migraine pathogenesis (reviewed in [21]). Accordingly, migraine is the first cause of disability among young women [8]. It is arbitrary divided into two forms: episodic migraine when the number of days of headache attacks is less than 15 per month or chronic migraine when the attacks occur for 15 or more days per month [22]. Episodic migraine may transform into chronic migraine in the process of chronification [23]. There are more classifications of migraine, including its categorization into migraine with or without aura - sensory disturbances, such as flashes of light, blind spots, and other vision changes or tingling in hand or face [24]. The clinical pictures of migraine with and without aura are so diverse that some researchers suggest considering them as two different entities [25, 26].

The prevalence of migraine in the elderly (above 60 years old) is less than in younger individuals with both incidence and prevalence decreasing with age [27]. The clinical phenotype and symptoms of migraine in the elderly are different from that observed in younger patients, e.g., headaches are bilateral rather than hemispheric. Treatment options are complicated due to many comorbidities and polypharmacy in this group of migraine patients. In addition, there are much fewer publications on migraine in the elderly than in younger patients.

Migraine is a complex disease with both environmental/lifestyle and genetic/epigenetic factors involved in its pathogenesis. Genetic studies performed on rare monogenic migraine subtypes, including familial hemiplegic migraine, identified causal mutations in the calcium voltage-gated channel subunit alpha1 A (*CACNA1A*), ATPase Na+/K+ transporting subunit alpha 2 (*ATP1A2*) and sodium voltage-gated channel alpha subunit 1 (*SCN1A*) genes that are involved in transport of ions at synapses and glutamatergic transmission (reviewed in [21]). A recent study by the International Headache Genetics Consortium (IHGC) with over 100 000 migraine patients identified 123 loci that conferred risk for development of migraine [28]. These loci contain variants of genes implicated in vascular function, neurotransmission at glutamatergic synapses, synapse development and plasticity, nitric oxide signaling, and metalloproteinases activity [29]. In addition, variants of genes encoding effective therapeutic targets in migraine: CGRP and 5-hydroxytryptamine (serotonin) receptor 1F (5HT-1F), were associated with migraine. Environmental/lifestyle factors of migraine pathogenesis, which are considered as the disease triggers, are stress or subsequent relaxation, fasting or skipping a meal, too much or too little sleep, ovarian hormone changes, including menstruation and oral contraception, weather changes, physical exercise, alcohol, odors, including cigarette smoke, intense light and noise as well as many diet components [30, 31].

Although precise mechanisms of migraine pathogenesis are not known, it is accepted that the trigeminal nerve, the main sensory nerve innervating the head, plays a key role [32]. The sensitization of trigeminal ganglion (TG) neurons is involved in migraine and other primary headache disorders, but the type of cells and pattern of expression of genes implicated in migraine pathogenesis are largely unknown. Recently, an integrated single-cell transcriptomics and epigenomics analysis identified mouse and human neuronal and non-neuronal TG cell types that were transcriptionally active in migraine [33]. The non-neural cells were satellite glia, myelinating and non-myelinating Schwann cells, ﬁbroblasts, immune cells, and vascular endothelial cells. Also, TG cell types that were implicated in migraine by human genetic variation were identified. The authors compared the atlases of transcriptionally active cells within TG from human and mice, indicating some important differences between them, including the expression of the calcitonin related polypeptide alpha (*CALCA*) gene encoding CGRP in pruriceptors in humans and lack of such expression in mice. Targeting CGRP and its receptor by antibodies and antagonists revolutionized migraine treatment [34]. This and other differences should be considered in the use of a mouse model of human migraine.

Migraine prevalence with age displays somehow unusual age dependence as it peaks between the ages of 20 and 40 years, both in women and men [35]. Therefore, it is rational to take a closer look at the dependence of migraine prevalence and onset on age.

## 3. Chronological aging

Age-specific clinical differences in migraineurs were shown in a large, population-based study [36]. A decrease with age was shown in such variables as stress as a migraine trigger, photophobia, phonophobia, dizziness, throbbing, pressure, stabbing, and being forced to sleep or rest with headache. On the other hand, an increase with age were seen in alcohol, smoke and neck pain triggers, neck pain location, and running of the nose/tearing of the eyes. Older adults reported less intense migraine symptoms.

Although migraine in the elderly is a major neurological disease, its prevalence tends to decrease with age (reviewed in [27]). The phenotype of migraine cases in the elderly is also different from that in younger patients - migraine occurs more frequently in the bilateral form in the former than in the latter. This provokes the question on the role of aging in migraine pathogenesis and possible influence of migraine on the process of biological aging. The answer to these questions is especially important in the context of the worldwide increase in life expectancy.

As mentioned, the average prevalence of migraine in women is 2-3 times greater than in men. However, the percentage of males with migraine decreased in the course from childhood to adulthood. Moreover, some migraine features in females, such as duration of headache and unilateral pain as well as aura-associated symptoms, such as noise and light sensitivity, increased with age [37]. Sensory sensitivities and nausea and vomiting were reported to reduce in older patients, while autonomic symptoms, such as facial flush and tachycardia were observed to increase in that group of patients [27]. These and other differences indicate the need of different therapeutic strategies for women, children, young adults, and the elderly. However, the question about the significance of true aging in migraine remains unanswered.

Late-life migraine accompaniments (LLMA) were for the first time described in older adults mimicking transient neurological episodes and other serious conditions [38]. LLMA are observed in migraine with aura but without headaches (silent migraine). Visual symptoms dominate in clinical presentation of LLMA along with sensory, aphasic, and motor indicators. This is another example illustrating a complex relationship between migraine and aging.

## 4. Brain aging

The brain is particularly sensitive to aging, but some elderly individuals show an exceptionally high intellectual ability, despite physical impairment typical for their chronological age. Therefore, the brain may be an example of not only differences between chronological and biological aging, but also organ-specific aging. The term “normal aging” or “healthy aging” is also applied to the brain, suggesting that it may age without any disease sign, but recent research suggests that the aged brain is a pathological state per se (reviewed in [39]). On average, ten percent of individuals at 65 years or older displayed age-related pathological changes that can be linked to Alzheimer disease (AD) [40]. Brain aging alone may impair inductive reasoning, spatial orientation, verbal memory and affect prognosis in stroke [41, 42].

Morphologically, brain aging is primarily characterized by brain volume loss, cortical thinning, white matter degradation, loss of gyrification, and ventricular enlargement. From the pathophysiological point of view, brain aging is linked with shrinking of neurons, dendritic degeneration, demyelination, small vessel disease, decreased metabolic rate, microglial activation, gray and white matter volume changes and white matter lesions (reviewed in [43]). On the cellular level, glial cells, astrocytes and microglia, play a major role in brain aging mechanisms. Astrocytes support nutrients intake, ion transport, exert an anti-inflammatory effect and assist integrity of the blood-brain barrier (BBB) [44]. However, these features of astrocytes are weakened and lost during aging and instead these cells release toxic factors killing neurons and oligodendrocytes [45]. Microglia are thought as a leader of white matter aging, acting as a modulator of diverse glial cells and phagocytosing white matter-derived myelin debris (reviewed in [39]). Single-cell RNA sequencing from white and gray matter allowed for the identification of white matter-associated microglia (WAMs), which partly bear disease-associated microglia (DAM) gene signature and are characterized by the activation of genes involved in phagocytic activity and lipid metabolism [46]. WAMs may be a kind of protective response needed to remove degenerated myelin accumulating during brain aging and disease. However, aging microglia show impaired phagocytic function causing accumulation of lipid metabolites and further degeneration of myelin sheath.

This is not the purpose of this work to explore general aspects of brain aging - this subject is presented in several excellent recent reviews, e.g., [39, 43, 47]. We try to relate some aspects of migraine pathogenesis to brain aging hallmarks. However, an association of migraine with an impairment in cognitive ability linked with aging brain, will be here considered in a broader context of cognitive and social aging in further sections.

As indicated, brain aging is associated with brain lesions, especially white matter damage [43]. For a long time, migraine has been considered not to have long-term consequences to the brain. However, several studies suggest that this may not be a universal case, but there are some conflicting results. An association between migraine occurrence and brain lesions risk was observed in a cross-sectional magnetic resonance imaging (MRI) study in an already existing population-based sample of adults with migraine and controls with not a headache history, the Cerebral Abnormalities in Migraine, an Epidemiological Risk Analysis (CAMERA) study [48]. Migraineurs with aura were reported to predominantly have such lesions as subclinical infarcts in the posterior circulation. Migraineurs had brainstem hyperintense lesions and female migraineurs were at increased risk of white matter lesions. However, the CAMERA-2 study found no association between deep WML load and change in cognitive features [49]. Furthermore, longitudinal studies showed no association between a history of migraine and an increased risk of dementia. Several subsequent works reported an association between migraine and brain lesions, but they neither showed nor suggested brain aging to mediate these changes [50]. Instead, several other reasons of brain lesions, especially in white matter, are suggested, including ischemia, neuroinflammation and endothelial dysfunction in the generation of spreading depolarization [51]. Therefore, it is difficult to assess how migraine is affected by brain aging and vice-versa and this is an important question which should be addressed in further research.

## 5. Social aging

Social aging is a term used to describe changes across the lifespan in social interactions and behavior in group-living organisms to cope with socio-environmental challenges [52]. The changes across the lifespan, although relevant to migraine, are difficult to analyze in migraine patients. Therefore, lifelong risk factors for accelerating aging are usually analyzed for their association with migraine. In humans, the social hallmarks of aging are low lifetime socioeconomic status, adversity in childhood and adulthood, being a member of a minority group, adverse health behaviors, and adverse psychological states [14]. Such hallmarks of aging were investigated as potential risk factors in migraine.

Low socioeconomic status (SES) was recognized as a risk factor in migraine and a factor aggravating its course. First of all, low SES can hinder and delay patient access to migraine treatment and result in poorer outcomes as shown in the Chronic Migraine Epidemiology and Outcomes Study [53]. An analysis of data from the American Migraine Prevalence and Prevention Study showed that both women and men with lower household (HH) income, reliably reported a higher symptom severity during acute migraine attacks, greater interictal symptom burden, and more serious functional impairment from their migraine symptoms [54]. The observed differences were not explained by race and other confounders. A higher migraine incidence rate at the lower HH income for both females and males were observed. An increase in prevalence with decreased HH income is typical not only for migraine. However, migraine remission rates did not depend on HH income. The obtained results agree with the social causation hypothesis [55]. Once initiated, migraine remission is independent of HH income. Therefore, onset and remission of migraine may have etiologically distinct causes. Although HH income not always positively correlates with SES, it can be considered as its good hallmark.

What has SES to do with aging? This is somehow puzzling as SES may be determined in an objective or a subjective way. In childhood and adolescence SES usually strongly depends on parents. It was shown that HH income did not have any significant effects on migraine prevalence in adolescents with family history of migraine [56]. The authors interpreted such results because of higher biological predisposition to migraine that might overwhelm any influence of HH income. This conception was confirmed by the results showing a positive correlation between migraine prevalence and HH income in adolescents without a family history of migraine. Altogether those results suggest social causation rather than social selection and indicate the need to study environmental risk factors related to HH income and migraine.

SES plays an important role in the migraine treatment and prevention, especially with anti-CGRP drugs that are still relatively expensive. However, it should be noted that migraine may influence SES, especially in education and employment. Also, migraine risk factors, such as smoking, or alcohol drinking may affect SES. On average, there is a dependence of SES on chronological aging, but more importantly some reports claim that it may influence the rate of biological aging [57, 58]. Again, on average, SES increases with age until retirement, but in many cases high professional activity and the highest income in gained around 40s. Therefore, time dependences of SES and migraine might have something in common.

If we assume that SES is timely correlated with the prevalence of migraine, the difference between sexes requires further explanations. An increased risk of migraine for low educated, retired, and unemployed individuals as well as smokers was shown in a large population twin-based Danish study [59]. Men displayed a higher migraine risk when they performed a heavy physical work, overused alcohol or were overweighted or obese (BMI > 25). A decreased risk of migraine for men compared to women was observed in individuals who were low educated, unemployed or studying. Using the data from the Korean Headache Survey, an influence of SES, especially living in rural areas, on migraine prevalence was shown in women, but not in men [60]. That study extended on Asia our knowledge on the role of SES on migraine prevalence, as majority of such studies were performed in Europe and the US. It also provided similar results for tension-type headache (TTH). It was observed that social determinants of health were associated with migraine prevalence both in men and women, but physical activity as an intervention factor in migraine seemed to be especially significant in women [61]. It was observed in a large cohort study, the Women Health Study, that women with low SES, expressed as a low HH income and a low measure of education, had an increased risk of all types of headaches, including migraine headache [62].

There are other aspects of social aging that are reported to be associated with migraine. It was hypothesized that insecure attachment was overexpressed in migraine patients and these patients had less social support as compared with general population [63]. This hypothesis was verified in a study with 101 consecutive patients evaluated with a battery of tests and questionnaires. An overrepresentation of insecure attachment styles was observed in migraine patients who had less social support than the general population, both in the fraction providing support and the level of satisfaction. However, aging was not considered as a confounding factor in that study, but the results confirm a possible role of social support in migraine pathogenesis. In a cross-sectional study with 37 migraine patients and 40 controls, it was shown that social support scores were negatively correlated with depression and anxiety scores. Multidimensional Scale of Perceived Social Support score was lower in migraine patients than controls. These studies suggest that social support may play a role in decreasing migraine attacks as it was found to affect the anxiety and depression scores of patients with migraine. In a similar study, assessing the role of work social support in migraine among 607 Chinese bank workers, it was shown that high social support in workplace was associated with a 74% reduction of chance to be affected by migraine [64]. Co-worker support had the greatest protective effect against migraine among six aspects of workplace support. Studies on work-associated and migraine performed in 94 consecutive subjects recruited in the outpatient clinic in Spain revealed that the MIDAS (Migraine Disability Assessment) score for both chronic and episodic migraine was inversely proportional to personal accomplishment at work [65]. Research on 900 nursing staffers in a tertiary medical center in Taiwan showed that 222 had migraine, 104 - tension headache, 37 - mixed migraine and tension headache, and 11 had other forms of headaches [66]. Headache sufferers had more stress at work than non-headache sufferers. The youngest and least experienced of the nursing staff, the unmarried, and those with a lower level of education had a higher level of stress, which illustrates an important role of social aging in migraine. Studies on 1076 workers from companies operating in different sectors showed that almost half of them suffered from headaches, which were associated with female gender, recent bereavement, intrusive leadership, and sleep problems [67]. A large cohort Brazilian study showed that migraine was associated with low job control, high job demands and low social support [68]. Job control was more strongly associated with migraine in women. In summary, stress at work is often reported to be associated with migraine, supporting the thesis that social aging may be an important contributor to migraine pathogenesis, but most of these works did not consider chronological aging as a confounding factor. Retirement is a period in life when an individual is relieved from stress work but may face other kinds of stress. Studies on the French GAZEL cohorts showed that statutory retirement was followed by improved sleep and lower rates of self-rated suboptimal health [69, 70]. An 11-13% reduction in headache prevalence was observed during pre- and postretirement in a study with nearly 16 thousand participants [70]. However, the maximal reduction in headache, 46%, was observed during the retirement transition. In absolute terms, such reduction was greater among persons with high work stress or persons with stress-prone personality than other participants [71]. Therefore, reduction of migraine prevalence with retirement supports thesis that social aging may be important in migraine pathogenesis and explains, at least in part, why migraine prevalence decreases with chronological aging.

In summary, social aging may influence onset and the course of migraine but it has not any effect on the disease remission. Different socioeconomic status of women and men as well as difference in the work and lifestyle, may contribute to differences in migraine prevalence between women and men. Biological susceptibility to migraine may overwhelm the effect of social aging in migraine pathogenesis.

## 6. Stem cell exhaustion and cellular senescence

Stem cells play an important role in organismal aging and stem cell exhaustion is a well-established molecular marker of aging [10]. Resident stem cells can be found in the CNS within specific niches in which they maintain their capacity to renew the populations of neurons, astrocytes and oligodendrocytes [72]. Endothelial progenitor cells (EPCs) are of interest to study in migraine due to its association with an increased risk of cardiovascular and cerebrovascular diseases [73, 74]. The number of circulating EPCs is a surrogate biological marker for endothelial function [75].

Cellular senescence was originally restricted to an irreversible inhibition of cellular division, but now it is seen as a process associated with numerous changes in the affected cell and its vicinity, including other cells. Besides replicative senescence, resulting from the replication end problem, senescence may be prematurely induced by a stress that may also affect stem cells, both during producing progenitors and in their resting state in a niche. The relationship between stem cell exhaustion and cellular senescence is complex, may be mediated by many factors and is not completely known (reviewed in [76]). Besides a simple implication that cellular senescence contributes to stem cell exhaustion, both phenomena likely interplay to drive the process of aging [77]. Senescent cells develop the senescence-associated secretory phenotype (SASP) featured by the overproduction of growth factors, proteases, pro-inflammatory cytokines and extracellular vesicles, which may act in autocrine or paracrine manner [78]. Cellular senescence can contribute to physiological aging, but its connection with pathological aging and diseases, although presented in many reports, is not fully known [16, 79]. The presence of a small fraction of senescent cells in early life is favorable for further development and homeostasis, but accumulation of senescent cells in advanced age may be detrimental [80].

The active products released by senescent cells may induce low-grade inflammation important in the pathogenesis of migraine [81, 82]. A decreased number and reduced functionality of circulating EPCs was observed in migraine patients with and without aura as compared with subjects with TTH and without headaches [83]. Regression analysis showed that migraine was a predictor for EPCs number. Functionally, EPCs from migraine patients displayed decreased migratory capacity and increased cellular senescence compared with EPCs from TTH or subjects without headaches. Therefore, migraine may be associated with a decrease in the stem cells population needed to replenish cells that lost their functionality. These results were generally confirmed and extended in a subsequent study in which a reduced number of EPCs was observed in episodic migraine patients both during headache attacks and in interictal periods [84]. That study also showed that the number of EPCs decreased as migraine progressed in time.

These studies also suggest therapeutic use of stem cells in migraine. ClinicalTrails.gov provides information on one clinical trial with autologous mesenchymal stem cell preparation for treatment of refractory migraine (https://clinicaltrials.gov/ct2/show/NCT04064879?term=stem+cell&cond=Migraine&draw=2&rank=1). The rationale for the use of mesenchymal stem cells to counteract reduced number of endothelial cells was that the former might differentiate into the latter and both kinds of cell might crosstalk in tissue regeneration [85, 86]. However, this phase I study started in August 2018, has been suspended. Prior to that study, it was shown that autologous adipose-derived stromal vascular fraction, rich in mesenchymal stromal cells, injected in patients with chronic refractory migraine improved their state [87]. Seven out of nine migraine patients showed an improvement in MIDAS score, but only in two of them such improvement was significant. The authors explained their results by the potential of mesenchymal stromal cells to target migraine-related neurogenic inflammation, but that speculation requires further research. That study was some kind of extension of an earlier study showing an improvement in a few women suffering from migraine and TTH after administration of stromal vascular fraction [88].

The involvement of CGRP, a key target in migraine therapy, in stem cell maintenance and functions was reported in many studies (e.g., [89-91]), but results of these studies cannot be directly related to migraine. In general, many reports show a positive role of CGRP in stem cell maintenance, first of all through the management of the stem cell niche [92-94]. This may be related to migraine as a protective mechanism against stress-related energy deficit in the brain and antioxidant properties of CGRP that will be discussed further [95].

SASP may induce signaling pathways that produce reactive oxygen and nitrogen species (RONS) [96]. DNA damage response (DDR), an evolutionary developed cellular reaction to DNA damage, induces cellular senescence to stop cell cycle with highly damaged DNA [97]. DNA damage may be considered as a prototype to induce cellular senescence. The efficacy of DDR decreases with age, which is important in the pathogenesis of many diseases, including cancer [98]. Recently we suggested that DDR may be compromised in migraine, but further studies including chronological age as a confounding factor are needed to address that issue [99].

In summary, vascular aspects of migraine may be associated with exhaustion of endothelial stem cells with aging and progenitors of mesenchymal stem cells may target neurogenic inflammation associated with migraine. A low-grade inflammation may be induced by senescence associated secretory phenotype along with oxidative stress and CGRP may be involved in stem cell maintenance by counteracting oxidative stress. Therefore, migraine may correlate with stem cell exhaustion and cellular senescence increasing with aging.

## 7. Epigenetic aging

Epigenetic regulation of gene expression finally decides about the phenotype of a cell or organism. It includes DNA methylation, posttranslational histone modifications and the action of non-coding RNAs. The epigenetic pattern of the genome (epigenome) is partly perpetuated from one generation to the next, but this process is not so precise and tightly regulated as DNA replication. The epigenetic profile or epigenome, understood as the complete set of its epigenetic modifications and associated non-coding RNAs, changes with age, but at present the precise nature and the course of these changes are not known [18]. A partial explanation of such change may be an observation that the activity of enzymes responsible for the maintenance of the profile declines with age, but such activity may depend on the expression of genes encoding these enzymes, which in turn, depends on their epigenetic profile.

Historically, epigenetic aging is a term that was used to correlate changes in DNA methylation with chronological or biological aging [100]. It was observed that the number of the CpG dinucleotides in CpG islands with methylated cytosine changed with age [100-102]. Consequently, the term “epigenetic clock” was introduced to predict the chronological age of an individual from the methylation status of CpG dinucleotides and accordingly the term “epigenetic aging” was also introduced. Currently, many CpG sites in various tissues are considered to correlate with biological rather than chronological aging (reviewed in [17]). It is not the purpose of this review to discuss aspects of epigenetic clocks and epigenetic aging, especially since we think that these terms are somehow overused as they are limited to DNA methylation, which is a single component of the epigenetic profile. Moreover, association of DNA methylation with brain age may not be so strong as it is reported when properly adjusted for confounding factors. Over four hundred thousand CpG sites were interrogated across the genome of postmortem brain from 740 individuals aged 66-108 years [103]. An association of DNA methylation with aging was observed in 8156 CpG sites, but it decreased to 4263 after adjustment for sex, six common age-related neuropathologies, and cell type mixing proportion. Moreover, the number of methylated sites in DNA may not be a measure of the importance of methylation in aging as it is not known whether aging is a mass effect of many small changes.

Migraine chronification was shown to accompany changes in DNA methylation profile in an 11-year retrospective, genome-wide association study (GWAS) [104]. The most pronounced changes occurred in the SH2 domain containing 5 (*SH2D5*) and neuronal pentraxin 2 (*NPTX2*) genes. These results were confirmed in a subsequent, case-control study [105]. The products of both genes are involved in the control of synaptic plasticity, a process that is important for migraine pathogenesis [106]. SH2D5 does so through its involvement in the inhibition of excitatory synapse formation through the control of Rac (rac family small GTPase)-GTP level and NPTX2 - trough binding and clustering of the glutamatergic AMPA (α-amino-3-hydroxy-5-methyl-4-isoxazolepropionic acid) receptor [107, 108]. SH2D5 was not evidenced to be directly involved in aging, but there are reports on the involvement of reduced NPTX2 in cognitive dysfunctions, especially in AD [109]. The connection of cognitive function with aging is presented in the subsequent section.

Medication overuse headache (MOH) is a syndrome that in many cases is secondary to chronic migraine and in fact, there are not reliable markers to distinguish these two diseases [110]. It was observed that the catechol-O-methyltransferase (*COMT*) gene was hypermethylated in MOH patients [111]. Variability of the *COMT* gene was associated with changes in processes that can be related to aging, including decline in cognitive functions [112-114]. However, the relationship between genetic and epigenetic profile of a specific gene, if any, is not known, so these studies require validation by further research to be related to aging in migraine.

No differences were observed in epigenetic aging, measured by five different epigenetic clocks between episodic migraine and MOH patients as compared with individuals without headache [115]. Many parameters of epigenetic clock were investigated, but the study suffered from a relatively small number of subjects. Another problem was with chronological aging as a confounding factor, because a commonly used method for calculation introduces a bias in the final results. The authors used two methods of analysis to include chronological aging in their considerations.

Sixty-three differentially methylated regions (DMRs) rich in regulatory elements were identified close to some genes in another GWAS project in migraine [116]. These genes included members of the solute carrier family: solute carrier family 2 member 9 (*SLC2A9*), *SLC38A4* and *SLC6A5*. These genes were associated with aging in pathological conditions [117, 118]. Another gene, encoding diacylglycerol kinase gamma (DGKG) was associated with aging and it was dysregulated in colorectal cancer through DNA hypermethylation [119]. Remaining genes differentially methylated in migraine were kinesin family member 26A (*KIF26A*), dedicator of cytokinesis 6 (*DOCK6*), and complement factor D (*CFD*) that were reported to associate with various aspects of the aging process [120-122].

A higher migraine risk was reported in females with a low level of DNA methylation in the gene encoding receptor activity modifying protein 1 (*RAMP1*), which is a key receptor unit of CGRP [123]. The expression of receptor activity proteins in specific tissues was associated with rat aging [124].

Many studies explored epigenetic connections of CGRP in migraine (reviewed in [125]). Modifications of the epigenetic profile of the *CALCA* gene in migraine include DNA methylation, as the promoter of the gene has two CpG islands with two CpG sites that are hypomethylated in migraine patients [126]. Changes in histone modification pattern and effects of micro RNAs (miRNAs), circular RNAs, and long-coding RNAs (lncRNAs) on the *CALCA *gene were also related to migraine [127-130]. Conversely, CGRP can change the epigenetic profile of neuronal and glial cells as shown for its involvement in epigenetic regulation of neural cytoarchitecture changes related to migraine chronification mediated by histone deacetylase 6 (HDAC6) [131]. The fundamental role of CGRP in the transmission of neurogenic pain may be epigenetically regulated [132]. In line with this research, it was observed that reduced CGRP signaling led to lifespan extension in mice and roundworms (*C. elegans*) [133]. The longest-lived C57BL/6 male mouse reached an age of nearly 41 months. The longevity was at least in part due to decreased incidence of cancer. Therefore, reducing the growth factor signaling from CGRP may play the key role in longevity, but these results cannot be directly translated into humans, in which cardiovascular disease, not cancer, is the main cause of mortality, as CGRP may protect the endothelium. However, that study showed an improved energy expenditure and circadian rhythm when CGRP signaling was disrupted.

In summary, migraine may be associated with changes in the cellular epigenetic profile. These changes may affect genes that can be related to aging. However, at present it is difficult to decide whether these changes can be classified as “epigenetic aging”, especially since they are not limited to DNA methylation. Moreover, at present there are too few studies to specify a causal relationship between epigenetic aging and migraine.

## 8. Cognitive aging

Changes in cognitive functions are typical to an aging brain [134]. It is still an open question whether migraine may modulate such changes in cognitive functions or/and vice versa - whether impairment in cognitive functions may contribute to migraine pathogenesis. It seems especially important in the context of the association between migraine, all-causes dementia and AD [135-137].

Cognitive aging is a term introduced to underline mental changes occurring during chronological aging. It may be understood as a process of gradual, longitudinal changes in cognitive functions that are associated with aging [138]. Several studies suggest that strong headache attacks may impair cognitive functions (e.g., [139] for review). Furthermore, neuropsychological impairments in executive functions, memory and learning were shown to resume after headache attacks. Many studies point at an impairment in cognitive functions during migraine attacks, but the data on the interictal period are conflicting [140]. The specific cognitive domains that may be impaired in migraine are information processing speed, basic attention, executive functions, verbal and non-verbal memory and verbal skills.

Some studies, including the Rotterdam Study, suggest an improvement of cognitive functions in migraine patients [141]. It was shown that older (between 5th and 7th decades of life) individuals with migraine and non-migraine headaches were not at an increased risk of cognitive decline, cognitive impairment or dementia [142]. That study used a comprehensive neuropsychological battery of tests of memory, language and executive functions, repeated 5 years apart. It was observed that individuals with migraine presented more subjective cognitive complaints and depressive symptoms than non-headache controls. In the context of aging, it is interesting whether such a state is maintained for longer time. In general, it requires further research to determine whether cognitive aging contributes to migraine or migraine modulates cognitive aging. Also, the Epidemiology of Vascular Aging (EVA) study, provided negative results on the association between migraine and decline in cognitive functions [143].

Many more recent studies suggest a positive association between migraine and impairment in cognitive functions/dementia (reviewed in [137]). Not only all-cause dementia but also AD, were observed at a greater ratio in 65+ years old migraineurs than age-matched controls [144]. Similar results were obtained for women diagnosed in general practices in the United Kingdom [145]. A Danish national register-based follow-up study revealed that migraine was a midlife risk factor for dementia in later life [146]. A higher ratio of dementia was observed in individuals with migraine with aura than without aura. That study suggests that migraine may affect cognitive aging. A randomized, cross-sectional, within subject approach study showed that not only migraine occurrence, but also its duration and frequency of headache attacks were positively correlated with an impairment in cognitive functions [147].

Studies on the association between migraine and cognitive functions in children and adolescents provided some contradictory results, but certain studies suggest that children and adolescents affected by migraine may display specific cognitive deficits, such as impaired short and long-term verbal memories, speed processing information, and selective/divided attention [148, 149].

An association between chronological aging and a decline in headache days in the absence of any confounding cognitive pathology was observed [150]. Moreover, aging can be a weak predictive indicator of headache days across the cognitive spectrum. Further research may explain whether results of these observations represent a reporting bias due to dementia or are biologically relevant.

In summary, studies on the association between migraine and impairment in cognitive functions did not yield consisted results in general, but many recent data report an association between them. Certainly, migraine impairs cognitive functions, but several studies show that this may be a transient state, which resumes after cessation of the headache attacks and so it cannot be attributed to cognitive aging. However, the state of the brain was not investigated in those studies. Further studies might determine a possible causative relationship between migraine and cognitive aging.

## 9. Metabolic aging and oxidative stress

The rate of metabolism and the amount and composition of its by-products change with age and were considered as drivers of the aging process (reviewed in [151]). The term “metabolic aging” relates the rate of metabolism to chronological aging. Aging may be metabolically characterized by insulin resistance, changes in body composition and expression of genes involved in growth control. Regulation of metabolic processes in aging organisms may be linked with extension of longevity through nutritional intervention and lifestyle modifications, both of potential significance in migraine pathogenesis. Not only lifespan, but also healthspan can be modified by metabolic intervention [152]. Metabolic stress may be linked with inflammation, oxidative stress, mitochondrial dysfunction, endoplasmic reticulum stress, changes in the unfolded protein response, impairment in proteostasis and other effects that may modulate aging [153].

It was hypothesized that migraine may be an element of a defense mechanism to protect the organism against a metabolic challenge [154]. An immediate question is why some people experience migraine and the others do not, despite the same metabolic challenge, but the answer is still the same - some people may have a biological predisposition to migraine increasing their sensitivity to other biological factors and environmental/lifestyle influences.

Metabolic pathways involved in energy production in the brain are natural candidates to associate with migraine pathogenesis. Synthesis of ATP from glucose is the main pathway of brain energy production, which occurs in three key stages: glycolysis; the citric acid cycle also known as the Krebs cycle or tricarboxylic acid (TCA) cycle and oxidative phosphorylation [155]. Deficit in brain energy metabolism in migraineurs was reported in MRI studies showing a lower phosphocreatine to creatine ratio and an enhanced concentration of ADP as compared to non-migraineurs (reviewed in [156]). Energy deficit positively correlated with severity of headache attacks [157]. Mechanistically, brain energy deficit may be a source of oxidative stress, which may contribute to migraine pathogenesis [95].

It was suggested that insulin resistance was an adaptive response to migraine to increase energy supply to ameliorate its deficit in the migraineurs brain [158]. Conversely, the “neuroenergetic hypothesis” suggests that postprandial hypoglycemia may play a major role in the pathogenesis of episodic migraine and that brain insulin resistance, which is a key risk factor for Alzheimer disease, may significantly contribute to migraine chroniﬁcation [159, 160]. Reduced glucose metabolism, volume and energy metabolism in migraine are reported in several areas of brain, including insular cortex, parietal cortex, left prefrontal cortex and orbitofrontal cortex (reviewed in [159]).

**Figure 3.**
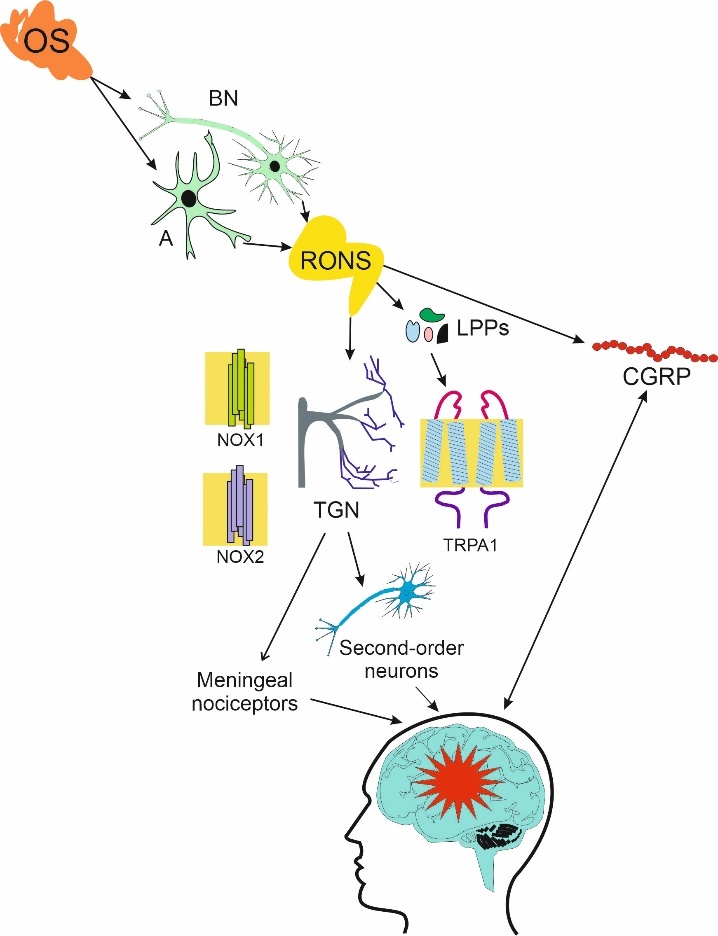
**Oxidative stress (OS) in the brain affects brain neurons (BNs) and astrocytes (As)**. OS may release reactive oxygen and nitrogen species (RONS) that may interact with transient receptor potential cation channel subfamily A member 1 (TRPA1) and take an oxidative stress-related signaling function to activate reduced nicotinamide adenine dinucleotide phosphate (NADPH) oxidase 1/2 (NOX1/2) in neurons of trigeminal nerve (TGN) and sensitize meningeal nociceptors and second-order TGN neurons, important in migraine, which is presented here as a red star. TRPA1 may induce the release of calcitonin gene related peptide (CGRP), strongly associated with migraine, from TGN neurons and dural tissue. Furthermore, RONS may also induce CGRP. Lipid peroxidation products (LPPs) are induced by RONS and may contribute to this cascade of events by TRPA1 stimulation.

The main reasons for the brain energy deficit in migraine are increased energy demand by hyperexcitable brain and lowered energy supply by impaired mitochondria [95]. The extent of energy production determines the amount of reduced nicotinamide adenine dinucleotide phosphate (NADPH), which, in turn, determines the activity of enzymes involved in antioxidant defense of brain neurons [161]. In addition, the expression of these enzymes is regulated by epigenetic modifications resulting from the expression and activity of proteins involved in the maintenance of the cellular epigenetic pattern. Mitochondrial NADPH is synthesized by enzymes involved in energy generation, including isocitrate dehydrogenase of the TCA cycle and nicotinamide nucleotide transhydrogenase (NNT), that depend on the Krebs cycle and oxidative phosphorylation [162]. However, when energy generation is impaired, these enzymes may consume antioxidants. Cortical spreading depolarization, an effect associated with migraine aura, induces a decrease in NADH (reduced nicotinamide adenine dinucleotide) level resulting in impairing antioxidant defense by NNT and overproduction of RONS [95]. The cellular antioxidant system consists of antioxidant enzymes, DNA repair proteins and small molecular weight antioxidants. All these elements are reported to progressively decline with aging [163].

**Figure 4.**
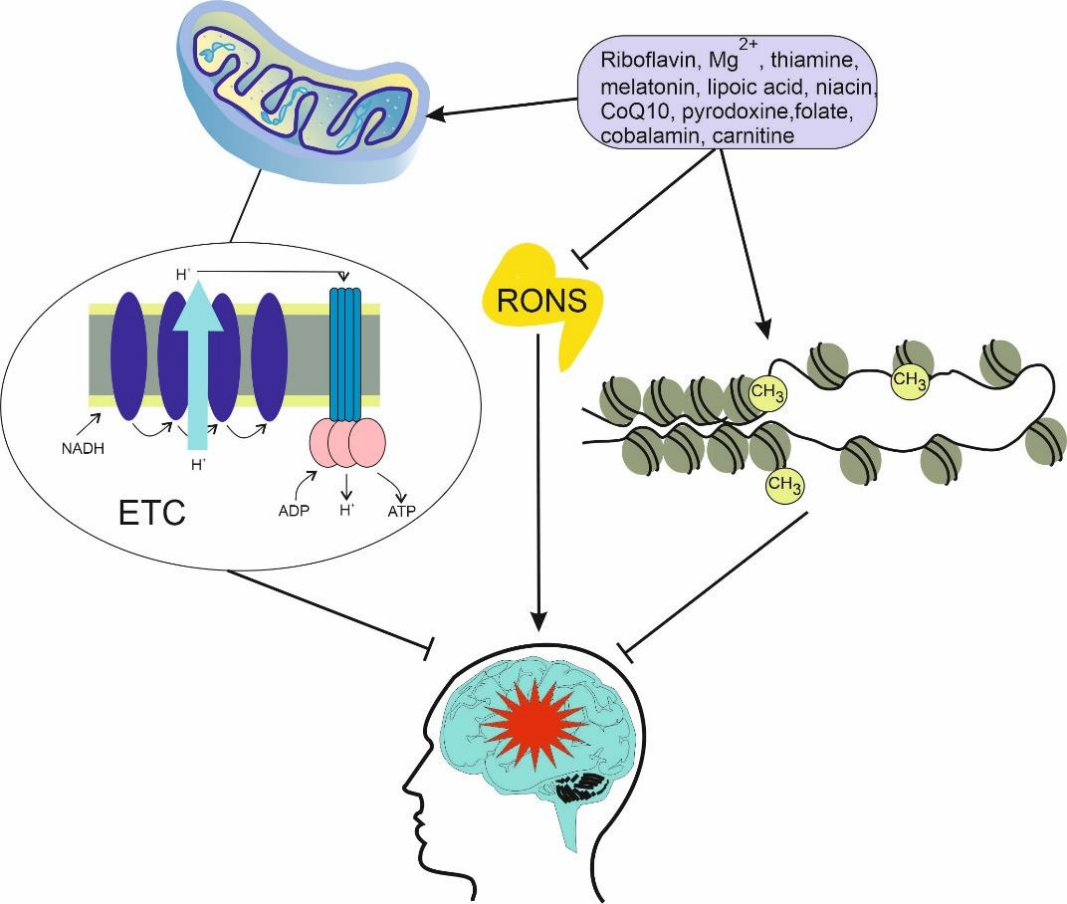
**Metabolic aging can be slowed down by some nutritional interventions resulting in preventing or ameliorating migraine, symbolized here as a red star**. These interventions may inhibit oxidative stress, represented by reactive oxygen and nitrogen species (RONS), improve mitochondrial metabolism and energy production through mitochondrial electron chain (ETC) and epigenetic changes in genes involved in energy metabolism, shown here as changes in the DNA methylation pattern and alteration in the chromatin structure. All these changes improve brain metabolism and compensate for brain energy deficit in migraine. Dietary interventions are represented by but not limited to some nutrients that were reported to display presented features. CoQ10 - coenzyme Q10, NADH - reduced nicotinamide adenine dinucleotide.

An acute oxidative stress in the brain may induce a migraine attack as a defense reaction against potentially harmful stress-related changes [164]. However, a causative relationship between oxidative stress and migraine has not been experimentally documented, but several pathways of association were suggested (reviewed in [95]). Hydrogen peroxide, and likely other RONS, may be released by neurons and astrocytes, trapped by transient receptor potential cation channel subfamily A member 1 (TRPA1) and transduced into a neural signal of oxidative stress through the activation of NADPH oxidase 1/2 (NOX1/2) in trigeminal ganglion neurons [165, 166] (Fig. 3). This may sensitize meningeal nociceptors and second-order trigeminal neurons, important in migraine-like headaches [167]. Activated TRPA1 induces CGRP release from TGN neurons and dura matter [168]. Hydrogen peroxide also stimulates CGRP release, supporting the role of oxidative stress in migraine pathogenesis [169]. Furthermore, oxidative stress results in enhanced levels of lipid peroxidation products that may stimulate TRPA1 [170]. Consequently, the activation of TRPA1 may result in pain signaling and neuroinflammation, typical for migraine attacks [167].

MRI studies showed that migraine was associated with deficit in brain energy during attack and interictal phase (reviewed in [171]). It was a consequence of decreased energy production and/or increased energy need in the migraine brain and might enhance oxidative stress, which could lower the threshold for migraine triggers. Conversely, energy deficit in the brain lowers the threshold for migraine triggers to induce attack to counteract oxidative stress [95]. Cortical spreading depolarization was reported to reduce NADH content causing a decrease in mitochondrial NADPH and decreasing antioxidant defense [172]. This initial decrease is followed by RONS overproduction by the mitochondrial electron transport chain (ETC) resulting from local tissue hypoxia and NADH binding. As mentioned, hydrogen peroxide and other RONS induced the release of CGRP, a trigger of delayed sensitization of trigeminal neurons. Therefore, enhanced level of CGRP in migraine may result from defense mechanisms against oxidative stress associated with the brain energy deficit.

Oxidative stress occurring in may result in enhanced DNA damage in neurons, astrocytes, and glial cells, and impaired DNA repair in these cells may disturb brain homeostasis. An enhanced oxidative stress in nontarget tissues was associated with migraine [173]. That study reported enhanced levels of 7,8-dihydro-8-oxoguanine (8-oxoG), a major DNA-derived oxidative stress marker, in plasma of migraine patients. 8-oxoG has been positively correlated with both premature and normal aging in many studies (e.g., [174-176]).

A ^1^H-NMR study showed that the serum metabolic profile of migraine patients was different from controls [177]. The most pronounced differences in serum concentrations were observed in lipids, amino acids and products of glucose metabolism. These metabolites may be considered as predictive markers for migraine as the diagnosis of this disease is based on interview and physical examination.

Although the important role of CGRP in many aspects of migraine pathogenesis is well established, recent research suggests that CGRP may not be a kind of migraine trigger that could induce a complete migraine attack (reviewed in [158]). Some studies report antioxidant and anti-inflammatory actions of CGRP [178-180]. Therefore, CGRP release associated with migraine attack may support the role of migraine as an adaptive response to oxidative stress associated with brain energy deficit related to metabolic changes.

Some kinds of nutritional interventions may slow down metabolic aging through several mechanisms, including an improvement of mitochondrial functions and epigenetic regulation, which are important in migraine [181, 182]. Therefore, such intervention may be considered to prevent migraine and slow down metabolic aging, but nutrients that may be migraine triggers should be avoided, even when they show anti-aging properties [183]. Some nutrients that can be considered to delay metabolic aging in migraine are riboflavin, thiamine, magnesium ions, niacin; carnitine; coenzyme Q10, melatonin, lipoic acid, pyridoxine, folate and cobalamin (Fig. 4).

In the absence of glucose stores, astrocytes produce ketone bodies from fatty acids in the process of ketogenesis, which can be induced by the ketogenic diet, featured by a low carbohydrates content [184]. Therefore, this kind of diet, which would supplement energy deficit caused by lowered glucose metabolism, is considered as a dietary intervention in migraine [185]. However, the application of this kind of diet in clinical practice does not show long term beneficial effects in migraineurs and cannot be excluded that some positive results are only due to weight or/and fat mass loss [186].

Metabolic syndrome (MetS) is a set of metabolic risk factors including enlarged waist circumference, dyslipidemia, systemic hypertension, and hyperglycemia. Several works show that MetS coexists with migraine, but the pathophysiological mechanisms of such comorbidity are not known (reviewed in [187]). Some studies show an impairment of insulin sensitivity in migraine, even in young, non-obese, non-diabetic, normotensive patients, but again, no mechanistic explanation of this relationship has been proposed and verified. In summary, migraine is associated with metabolic changes, but at present it is difficult to select specific metabolic pathways and metabolites that would be causative in migraine pathogenesis. Many established and putative migraine risk factors can be related to cellular, or organ metabolism and metabolic aging may decide whether these factors may play a causative role in migraine. However, energy metabolism is strictly associated with brain homeostasis and its sensitivity to migraine triggers. Therefore, effects linked with metabolic aging may play a particularly important role in migraine pathogenesis. Moreover, it should be established whether metabolic aging is associated directly with migraine or through its secondary effects, such as obesity or metabolic syndrome.

## 10. Outstanding questions

At present, several problems wait for the explanation in the relationship between migraine and different aspects of aging and several outstanding questions can be asked, including the following:

- What is the mechanism determining that some individuals are sensitive to migraine and the others are not?
- Why do people in the most productive age have the highest prevalence of migraine?
- Why the age characteristics of migraine in women is roughly the same as in men, despite the disease prevalence in the former is 2-3 times higher than in the latter?
- Is stress associated with social aging correlated with migraine prevalence?
- How does brain aging contribute to migraine susceptibility?
- How are migraine triggers and genetic/epigenetic predisposition related to ging?
- Does cognitive aging trigger migraine?
- Does metabolic aging relate directly to migraine or rather via its secondary effects?

All these questions are addressed in the concluding section.

## 11. Conclusions, Hypotheses and Perspectives

Migraine is the second worldwide cause of disability, and first among young women, with the total number of affected individuals about 1 billion [8]. There are many migraine classifications presenting many migraine types, including menstrual, ocular/retinal, silent, vestibular, abdominal, hemiplegic migraines, migraine with brainstem aura and others [188]. Some of them are per definition associated with their primary cause, e.g., menstrual migraine, but others may be triggered by various factors. Therefore, it is tempting to ask whether specific aspects of aging are associated with specific types of migraine. There are not data on this subject, and it would be too speculative to hypothesize about such association. In particular, we are not able to determine or even speculate what migraine type, if any, is behind social aging. We have tried to provide arguments that social aging stress positively correlates with migraine prevalence and therefore may contribute to its pathogenesis. All other aspects of aging may differentially influence migraine occurrence and progression, but the intensity of stress related to those aspects does not strongly correlate with migraine prevalence, but rather with chronological aging.

Migraine as a disease has a few unusual features as compared with other human disorders. Despite its common occurrence and serious consequences for affected individuals and a significant burden for societies, mechanisms of its pathogenesis are poorly known. We do not know why some people develop migraine while others do not. This poor knowledge is underlined by several reasons, including, but not limited to, a restricted accessibility of human CNS, in particular the brain, to research and a limited usefulness of laboratory animals to model human migraine. However, despite this very limited knowledge on migraine pathogenesis, its relatively effective treatment, the anti-CGRP therapy, has been applied. The next extraordinal feature of migraine is its 2-3 times higher prevalence in women than in men, but this difference increases to 3-4 times in women after puberty. Chromosome X-linked events, hormonal homeostasis and mitochondrial transmission are considered to elucidate this difference, but no a satisfactory explanation has been provided so far [189]. The next unusual characteristic of migraine is the dependence of its prevalence on age with the peak around the range 20-40 years. How do these facts influence our conclusions and the outstanding questions?

**Figure 5.**
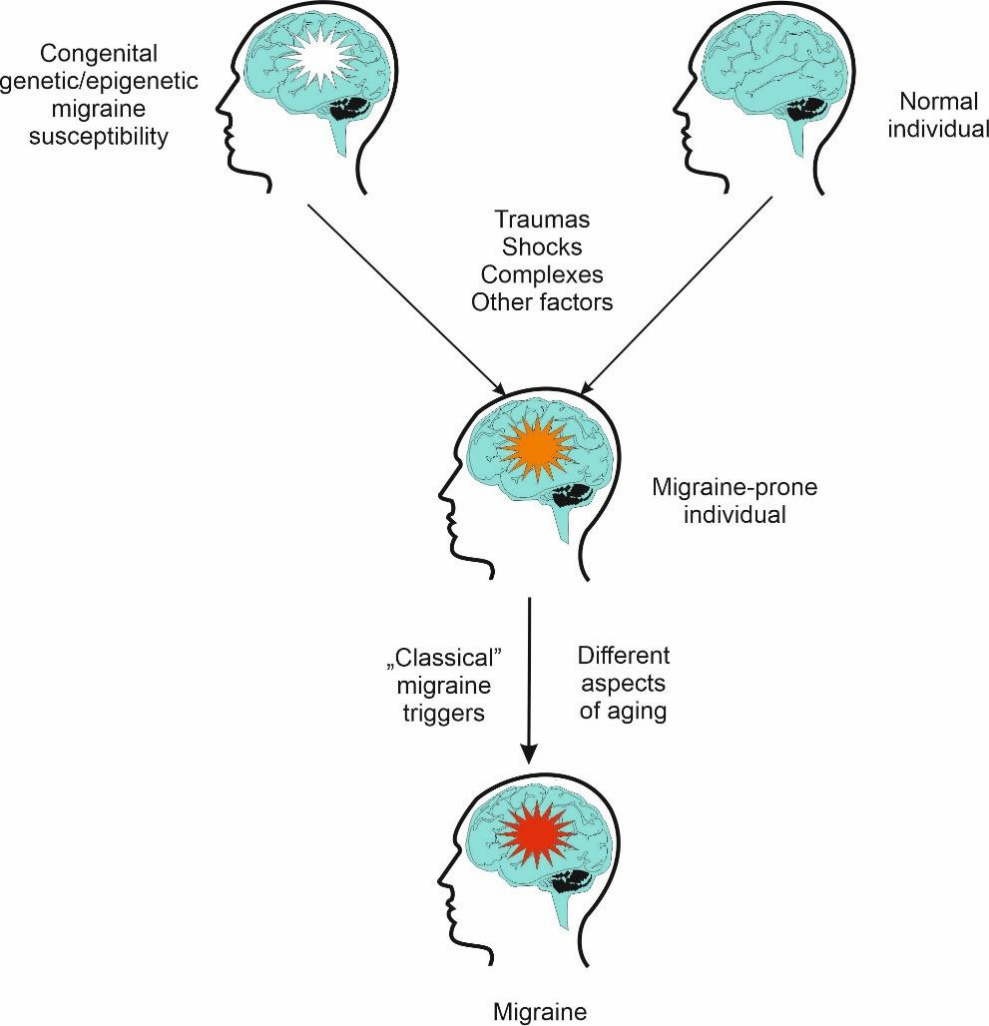
**Different aspects of aging may play a key role in a selective migraine susceptibility**. Migraine affects certain individuals, but for not fully known reasons, the others are not affected, although some of them are equally or even more exposed to “classical” migraine triggers (food component, odors, physiological complications etc.). We assume that migraines affect migraine-prone individuals. Such proneness can be congenital due to the changes in genetic and epigenetic constitution, symbolized here by a white star, or/and acquired due to traumatic events and other factors (orange star). Such migraine-prone person is susceptible for both “classical” migraine triggers and factors yielded by different aspects of aging presented in this work.

Firstly, all effects related to aging may play a role in migraine pathogenesis only in migraine-susceptible individuals. In other words, migraine is not a classical age-related disease. And who is migraine-susceptible? Not a “migraine gene” has been identified so far and there is not a perspective to do so. Surely, the term “migraine gene” is equally non-sense as e.g., “breast cancer gene”. Although several migraine susceptibility genetic loci have been identified, the genetic picture of migraine is far from complete. Moreover, such pictures must be supplemented by the epigenetic profile. We assume that migraine susceptibility is determined by genetic and epigenetic factors as the main components. What else could be important? Traumas, shocks, complexes and other events affecting physical and mental health acquired during childhood and later life may contribute to this susceptibility. Then, at least three concepts can be regarded. One considers migraine as a disease with strictly pathological aspects, the other - as a defense mechanism against stress affecting the brain. Moreover, migraine may be associated with reproductive or survival advantages from the evolutionary point of view [190, 191]. In each concept, the brain is in its center, but it is not the only one element important in migraine pathogenesis [192]. This migraine susceptibility (migraine background) weakly depends on chronological aging, as genetic/epigenetic constitution is its dominant component. A migraine-susceptible individual is prone to various influences, including “classical” migraine triggers and those associated with different aspects of aging, as we presented above (Fig. 5).

Which aspects of aging are the most important in migraine? If the migraine background is faintly dependent on age, these aspects should depend on chronological aging in a way similar to the relationship of migraine prevalence by age. We hypothesize that this is the stress associated with social aging, which may be mainly responsible for the induction and progression of migraine in migraine susceptible subjects. In many cases the most serious stress associated with everyday challenges occurs between 18 and 40 years as this time is linked to final and entrance exams, job hunting, professional challenges, starting a family, maternity/paternity and other stress-generating events. Before that time, a person is mainly dependent on his/her parents/guardians and after that stabilization time occurs in which many challenges no longer occur or are substantially weaker. The late period of life is very complex per se, and it is difficult to biologically link it to anything else than age-related impairment of life functions and diseases. There may be differences in facing these challenges by women and men, but it would be too speculative to relate them to migraine prevalence. Other aspects of aging may increase migraine susceptibility or directly induce migraine in migraine-susceptible individuals and metabolic aging may play an important role. This may follow from an important role of energy metabolism and significance of metabolism-related oxidative stress in brain homeostasis. Moreover, metabolic aging is strongly linked with other aspects of aging, including stem cell exhaustion, cellular senescence and epigenetic aging.

In this manuscript, we pointed out that migraine pathogenesis might contain two elements - inborn or acquired predisposition to migraine and aging related stress. It is tempting to consider the former in order to approach the answer to the question why some people develop migraine and others not, although they are exposed to the same migraine triggers. However, genetics of migraine, except rare familial hemiplegic migraine is poorly known and is supported by association rather than mechanistic studies [21, 193]. The epigenetics of migraine is even more poorly explored and still remains a “promising avenue” [125]. Therefore, there are not solid data to consider aging-related changes in the genetic and epigenetic profiles in migraine.

In a common understanding of the term age-dependence, “age” is considered as chronological age. Some mechanisms of migraine pathogenesis, underlined by physiological phenomena, are dependent on chronological age and may contribute to the age-dependence of migraine pathogenesis. Vasodilation and other vascular changes are progressively impaired with age and were considered to contribute to migraine pathogenesis, but nowadays they are treated as an epiphenomenon that is neither sufficient nor necessary to induce migraine [194-196]. As mentioned, migraine is associated with brain energy deficit, which can be translated into oxidative stress triggering a headache attack through oxidant-sensing nociceptive ion channels, including TRPA1, but aging is linked with a decrease in nicotinamide nucleotide transhydrogenase and therefore contribute to migraine occurrence [95, 164]. On the other hand, the brain may display a reduced sensitivity to oxidative stress when its basal levels rise with aging.

If we assume that the stress associated with social aging is the main age-related determinants of migraine occurrence and severity, the question is how remaining age-related factors influence migraine as their intensity increases with chronological age. This is an important problem in the elderly who accumulate consequences of such age-related factors, including migraine triggers to which the elderly is more susceptible than younger individuals. Cognitive aging, which may result in dementia and other mental disorders, is of a special significance not only in considering the mechanism behind migraine pathogenesis, but also in migraine diagnosis. Dementia may impede diagnosis of migraine as it is mainly based on self-reporting questionnaires. Other diseases typical for advanced age may be associated with non-migraine headaches and result in polypharmacy that may contribute to MOH, difficult to distinguish from migraine. Therefore, diagnostic criteria for migraine for the elderly may not be so adequate as for younger individuals and may require substantial modifications. Also, the prevalence of migraine in women after puberty is up to 4 times higher than in men, which provokes further questions about the age-dependence of migraine and diagnostic criteria in this disease.

In summary, many aspects of aging interwound in migraine pathogenesis, both molecular and cellular as well as social and cognitive. Migraine prevalence depends on aging in a way that fits the most social aging. However, effects attributed to that kind of aging cannot induce migraine in any person, but rather in individuals who inherited and/or acquired migraine susceptibility.
